# Research on the air supply adjustment technology of breath-following powered air-purifying respirators

**DOI:** 10.1038/s41598-023-39411-z

**Published:** 2023-07-27

**Authors:** Guangping Teng, Guoqing Shi, Jintuo Zhu, Caijun Zhao

**Affiliations:** 1 College of Safety Engineering, School of Safety Engineering, China University of Mining and Technology, Xuzhou, 221116 China; 2 School of Safety and Management Engineering, Hunan Institute of Technology, Hengyang, 421002 China; 3 State Key Laboratory of Coal Resources and Safe Mining, China University of Mining and Technology, Xuzhou, 221116 China

**Keywords:** Health occupations, Risk factors

## Abstract

In the hope of reducing the air supply flow of the powered air-purifying respirator (PAPR) and extending the service life of the filter, a breath-following powered air-purifying respirator (BF-PAPR) that can dynamically adjust the air supply flow according to the breathing flow is proposed. The BF-PAPR changes the air supply flow by adjusting the speed of the variable-frequency centrifugal fan according to the air velocity at the half mask outlet (*v*_*hm*_) monitored by the modular wind speed transmitter. In the study, the air supply flow adjustment model of the BF-PAPR is developed. It is found that the filtration resistance barely influences *v*_*hm*_. In addition, under the same mean inhalation flow, the minimum outlet air velocity increases first and then decreases with the increase of the duty cycle variation coefficient (λ), while the maximum outlet air velocity decreases first and then increases. Moreover, the minimum air supply flow of the BF-PAPR is achieved when the standard value of the air velocity is 13.4 m/s and the value of λ is 1. The BF-PAPR can reduce the air supply flow by 6.5%-8.6% and the energy consumption by approximately 20% compared with the PAPR, which is beneficial for reducing the usage cost and extending the continuous working time.

## Introduction

As the first option for dust control, engineering technology can fundamentally reduce harmful factors such as productive dust and inhalable particulate matter (PM_10_) that are likely to cause pneumoconiosis^1–4^. However, in some workplaces or jobs, engineering measures fail to effectively lower the dust concentration to below the national hygienic standard^5–9^. In this case, personal respiratory protective equipment becomes the last defense line to protect the health and safety of workers exposed to dust.

Self-inhalation air-purifying particle respirators (SIAPPRs), including disposable and replaceable particle respirators, are widely used at present^10,11^. However, SIAPPRs can raise the temperature in the respirator-covered area of users’ face^12–15^. Meanwhile, with the increase of attached dust on SIAPPRs, users suffer from greater respiratory resistance and feel suffocated, which has a negative impact on their lung function^16,17^. In addition, negative pressure is produced within SIAPPRs when users inhale, which may cause air leakage from them, thus weakening the protective effect^18–23^. Over the recent years, powered air-purifying respirators (PAPRs) have found wide application in multiple areas such as industrial production and public security. A PAPR is an air-purifying positive-pressure respirator that provides air flow to overcome component resistance by using an electric fan. It not only provides a better protective effect but also avoids such problems as rising face temperature and difficulty in breathing^24–26^.

In order to ensure a positive pressure state of PAPR masks, PAPRs generally choose the average of the peak inhalation flow at a moderate labor intensity as the standard for the minimum air supply flow. The U.S. PAPR standard stipulates that the minimum air supply flows for close-fitting and loose-fitting facepieces are 115 L/min and 170 L/min, respectively^27^. The minimum air supply flow is several times higher than the human inhalation flow, far exceeding the human inhalation demand^24^. However, current PAPRs adopt a constant air supply mode, and their air supply flows are large to ensure the protective performance, which leads to high energy consumption. Studies have shown that the pressure drop of the filter increases rapidly with the mass of dust deposition, which can potentially result in a reduction in air supply flow of PAPR, thereby degrading the respiratory protective performance^28,29^. In an environment where the dust concentration is 800 mg/m^3^, the filter needs to be replaced in even less than two hours to prevent the filtration resistance from exceeding the required value of PAPR^28^. Reducing the air supply flow is beneficial for extending the service life of the filter, thus reducing the usage cost.

In this study, a respirator that can dynamically adjust the air supply flow according to the breathing flow is proposed. Such a respirator can increase the air supply flow during inhalation and decrease the air supply flow during exhalation, thus achieving the purpose of minimizing the air supply flow while ensuring respiratory protection.

## Methods and materials

### Design of the breath-following powered air-purifying respirator

#### Single-chip microcontroller

Aiming at shortening the air flow movement distance and the time delay, a breath-following powered air-purifying respirator (BF-PAPR) that can be worn around the neck is designed. It mainly consists of a single-chip microcomputer, a variable-frequency centrifugal fan, a modular wind speed transmitter and a half mask. The wind speed sensor of the modular wind speed transmitter is set at the outlet of the half mask to monitor the change of air velocity at the outlet of the half mask ($v_{hm}$, hereafter referred to as the outlet air velocity) with the breathing flow in real time. The standard value of air velocity ($v_{s}$) is set in the BF-PAPR through the single-chip microcomputer. The fan speed is raised by the single-chip microcomputer to enlarge the air supply flow when $v_{hm}$ is lower than $v_{s}$, while it is lowered to decrease the air supply flow when $v_{hm}$ is higher than $v_{s}$. The STM32F103VET6 single-chip microcontroller (ST, Italy), which is selected based on functional requirements, incorporates the high-performance ARM® Cortex®-M3 32-bit RISC core operating at a 72 MHz frequency, high-speed embedded memories (512 KB Flash and 64 KB RAM), and an extensive range of enhanced I/Os and peripherals connected to two APB buses.

#### Variable-frequency centrifugal fan

**Figure 1:**
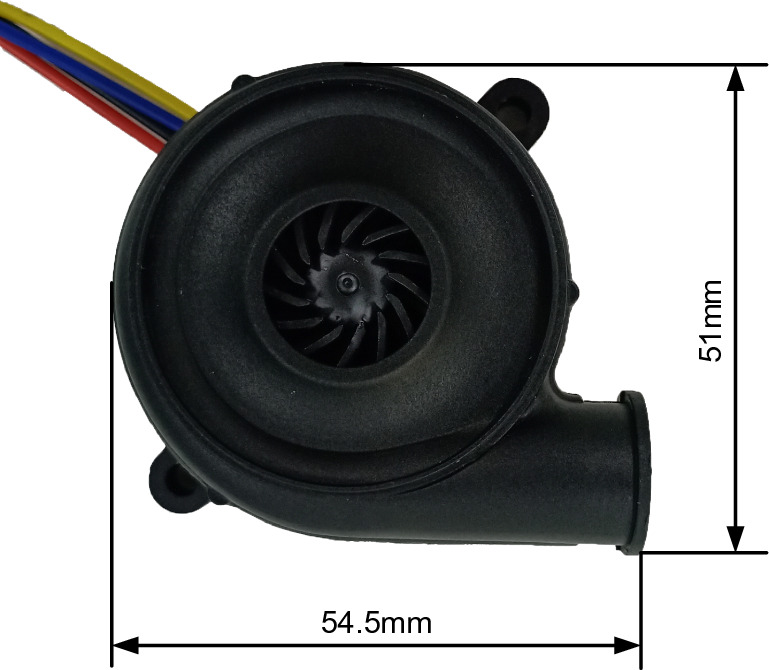
BA5025H12B-A variable-frequency centrifugal fan.

**Figure 2:**
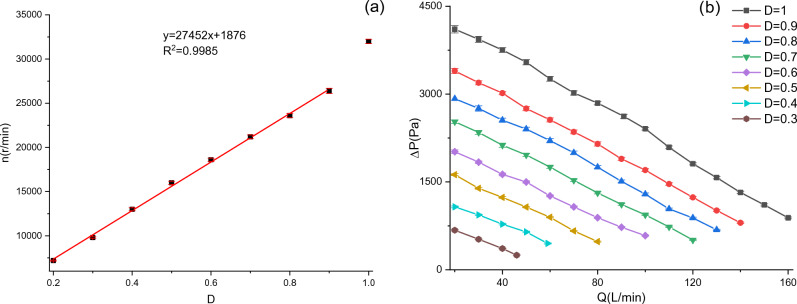
Performance of the variable-frequency centrifugal fan (**a**) relationship between duty ratio (*D*) and fan speed (*n*); (**b**) relationship between air pressure (*∆P*) and air flow (*Q*).

The BF-PAPR uses a BA5025H12B-A variable-frequency centrifugal fan (Shenzhen Tengkai Group Co., Ltd., Shenzhen, China) whose rated voltage is 12 V, maximum air flow is 300 L/min, and maximum air pressure is 4800 Pa (Fig. 1). Since the single-chip microcomputer controls the speed of the centrifugal fan by adjusting the pulse duty ratio, the speeds and performance curves of the centrifugal fan at different duty ratios were measured (Fig. 2). Error bars in Fig. 2 are standard deviations and are the same in all figures below. It can be seen from Fig. 2a that when the duty ratio is 0.2–0.9, the fan speed roughly increases linearly with the duty ratio, and linear fitting can be conducted; when the duty ratio is 0.9–1, the fan speed soars to 32,000 r/min. As can be seen in Fig. 2b, the air pressure at a duty cycle of 0.9 and an air flow of 115 L/min exceeds 1000 Pa. The standard specified by National Institute for Occupational Safety and Health (Respiratory Protective Devices: 42 CFR Part 84) requires a minimum air flow of 115 L/min for PAPRs equipped with a close-fitting facepiece^27^. Therefore, the variable-frequency centrifugal fan can meet the demand of the BF-PAPR.

#### Modular wind speed transmitter

**Figure 3:**
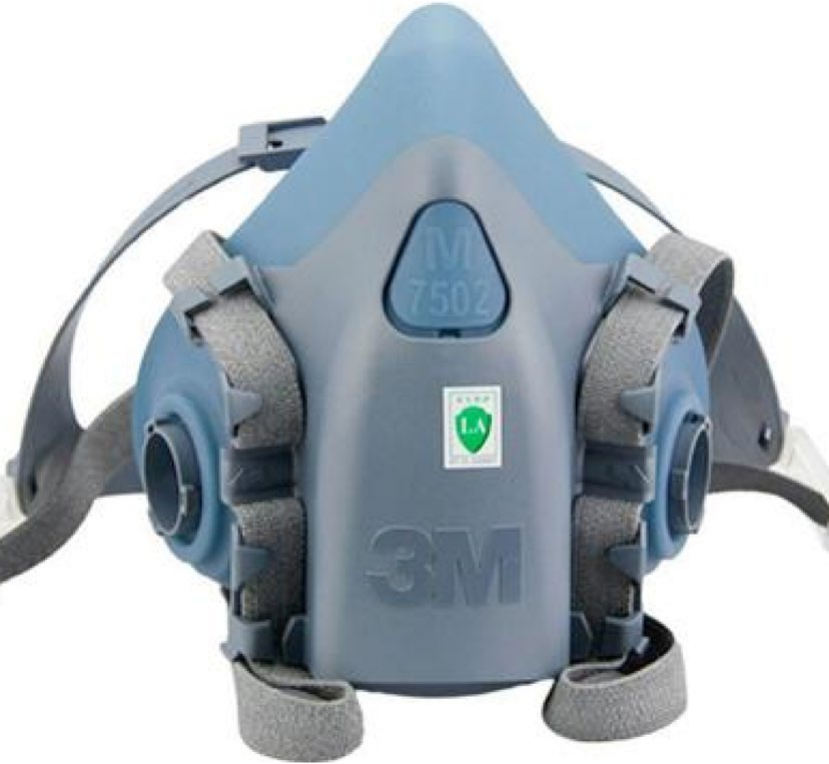
3 M 7502 half mask.

**Figure 4:**
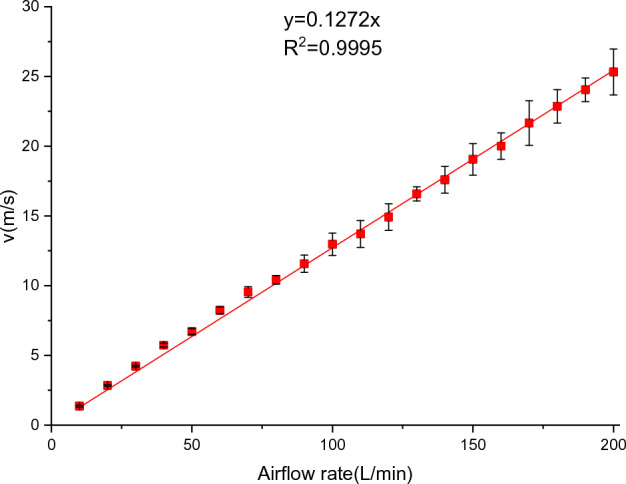
Relationship between air velocities and air flows at the half mask outlet.

The BF-PAPR uses a 3 M 7502 half mask and a W410F1 modular wind speed transmitter (Hangzhou QeaLy Technology Co., Ltd., Hangzhou, China). The half mask has air inlets on both sides (Fig. 3). The modular wind speed transmitter has a measuring range of 0–30 m/s and a resolution ratio of 0.01 m/s, and its wind speed sensor is fixed at the half mask outlet for real-time monitoring of the air velocity. The relationship between air flow and air velocity was investigated (Fig. 4). The investigation results reveal an approximate linear relationship between the measured air velocities and the air flows, and linear fitting was conducted. Through experimental studies, it has been found that a change of 10 L/min in the standard value of air flow at the half mask outlet is reasonable. To reduce the air supply flow of the BF-PAPR, 115, 105 and 95 L/min are selected as the standard values of air flow at the half mask outlet, and the corresponding standard values of air velocity are 14.6, 13.4, and 12.1 m/s, respectively.

### Air supply flow adjustment model of the BF-PAPR

The fan speed is adjusted according to the outlet air velocity during breath-following air supply, so as to stabilize the air velocity at around the standard value. Under the same working air resistance, the change of air flow of the fan is directly proportional to the speed ratio^30,31^:

1 $$\frac{{Q_{1} }}{{Q_{2} }}{ = }\frac{{n_{1} }}{{n_{2} }}$$

where $Q_{1}$ and $Q_{2}$ are the fan air flows, L/min; $n_{1}$ and $n_{2}$ are the fan speeds for air flows of $Q_{1}$ and $Q_{2}$, respectively, r/min. It can be seen from Fig. 4 that the outlet air velocity and the air flow are linearly correlated, so the speed variation is:

2 $$\Delta n_{2 - 1} = n_{2} - n_{1} = \frac{{\nu_{2} - v_{1} }}{{v_{1} }}n_{1}$$

Assuming that the outlet air velocity is $v_{0}$ and the fan speed is $n_{0}$ at $t_{0}$, then the variation of the fan speed when the outlet air velocity reaches the standard value of air velocity $v_{s}$ can be obtained by Eq. (3):

3 $$\Delta n^{\prime} = \frac{{\nu_{s} - v_{0} }}{{v_{0} }}n_{0}$$

In this experiment, the maximum speed of the fan is lower than 22,000 r/min, so the duty ratio (*D*) ranges from 0.2 to 0.9. Then, the variation of duty ratio ($\Delta D^{\prime}$) can be obtained from the fitting formula in Fig. 2a:

4 $$\Delta D^{\prime} = \frac{{v_{s} - v_{0} }}{{27452v_{0} }}n_{0}$$

Since it takes some time for the fan to change from the speed $n_{0}$ to the speed corresponding to the duty ratio of output signal from the single-chip microcomputer, the change rate of the fan speed is adjusted by changing the variation of duty ratio:

5 $$\Delta D = \lambda \frac{{v_{s} - v_{0} }}{{27452v_{0} }}n_{0}$$

where $\lambda$ is the duty cycle variation coefficient. Then, the duty ratio of output signal of the single-chip microcomputer is:

6 $$D{ = }D_{0} + \Delta D = D_{0} + \lambda \frac{{v_{s} - v_{0} }}{{27452v_{0} }}n_{0}$$

where $D$ and $D_{0}$ are the duty ratio of the pulse signal to be output by the single-chip microcomputer and the duty ratio at $t_{0}$, respectively.

### Experimental parameters

#### Breathing flow simulation

Cooper et al.^32^ found that the variation of human respiratory flow over time presents a roughly sinusoidal curve. In this experiment, a human breathing simulator was used to generate sinusoidally varying breathing flow. In this way, human breathing at different labor intensities could be simulated by adjusting the breathing rate and the mean inspiratory flow (MIF). According to ISO/TS 16976-1-2022^33^ and some previous studies^11,18,34–36^, the MIF was set as 15, 30, 45, 60, and 85 L/min in this experiment to simulate breathing flow in the case of a rising labor intensity, the corresponding breathing frequency being 10, 15, 20, 25, and 30 breaths/min, respectively.

#### Filtration resistance

In dust-containing air, the filtration resistance of the filter will gradually increase with the deposition of dust. Therefore, it is necessary to consider the effect of the increase in filtration resistance on the BF-PAPR. In this experiment, the change of resistance was simulated by increasing layers of filter media. At a constant air flow of 115 L/min, the average values of differential pressure were 294.8 ± 9.42, 591.2 ± 13.86, and 904.5 ± 18.27 Pa for 1, 2, and 3 layer(s) of filter media (*m*), respectively. In the breath-following air supply experiment, the influence of different filtration resistances on breath-following air supply was explored under the *m* values of 0, 1, 2, and 3, respectively.

### Respirator simulation experimental setup and procedure

**Figure 5:**
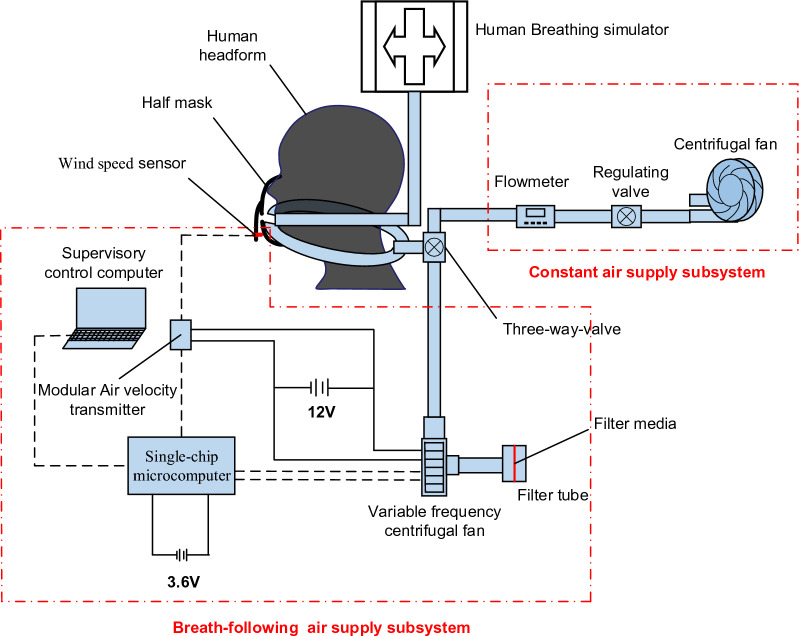
Schematic diagram of the experimental setup.

As shown in Fig. 5, the experimental setup is composed of two parts: a human breathing simulator system and a powered air supply system. The human breathing simulator system consists of a human breathing simulator, a human headform, and a half mask. The powered air supply system comprises a constant air supply subsystem and a breath-following air supply subsystem. The former is composed of a centrifugal fan, a regulating valve, and a flowmeter, while the latter is composed of a single-chip microcomputer (STM32F103VET6), a variable-frequency centrifugal fan (BA5025H12B-A), a filter pipe, filter media, a modular wind speed transmitter (W410F1), and a supervisory control computer. These two subsystems are switched through a three-way valve, and air pipes in the experimental setup is 20 mm in inner diameter. The filter pipe, which can hold the filter media, consists of 2 acrylic tubes with an inner diameter of 140 mm. The human breathing simulator can produce a sinusoidally varying breathing flow, adjust the breathing frequency and the MIF, and monitor the change of breathing flow in real time. To reduce the air supply distance of the variable-frequency centrifugal fan, the air pipe is connected with the three-way valve through both sides of the neck, and then directly linked to the variable-frequency centrifugal fan. The contact position between the half mask and the headform is sealed with glass glue to prevent air leakage.

The experimental procedure is described as follows: First, the human breathing simulator system was connected to the constant air supply subsystem by adjusting the three-way valve. Accordingly, the regulating valve was adjusted to set a flow rate of 115 L/min, and the MIF and frequency of the human breathing simulator were adjusted to simulate breathing flows under five different labor intensities. After the waveforms displayed in the monitoring computer and the human breathing simulator stabilized, data were recorded at a frequency of 20 Hz for 2 min.Subsequently, the human breathing simulator system was connected to the breath-following air supply subsystem. The experimental parameters are listed in Table 1. In the experiment, $v_{s}$ and λ were set by the single-chip microcomputer; the breathing flow was set by the human breathing simulator; and the filtration resistance was altered with the number of layers of filter media. Also, after the waveforms displayed in the monitoring computer and the human breathing simulator stabilized, data were recorded at a frequency of 20 Hz for 2 min. The above experiment was repeated three times to reduce errors.

**Table 1:** Setting of experimental parameters.

Experimental parameter	Experimentally set value
Standard value of air velocity $v_{s}$ (m/s)	14.6, 13.4, and 12.1
Duty cycle variation coefficient λ	0.5, 1, 1.5, and 2
Number of filter layers *m*	0, 1, 2, and 3
MIF (L/min)	15, 30, 45, 60, and 85

## Results and discussion

### Variations of fan speed and outlet air velocity of the BF-PAPR with breathing flow

**Figure 6:**
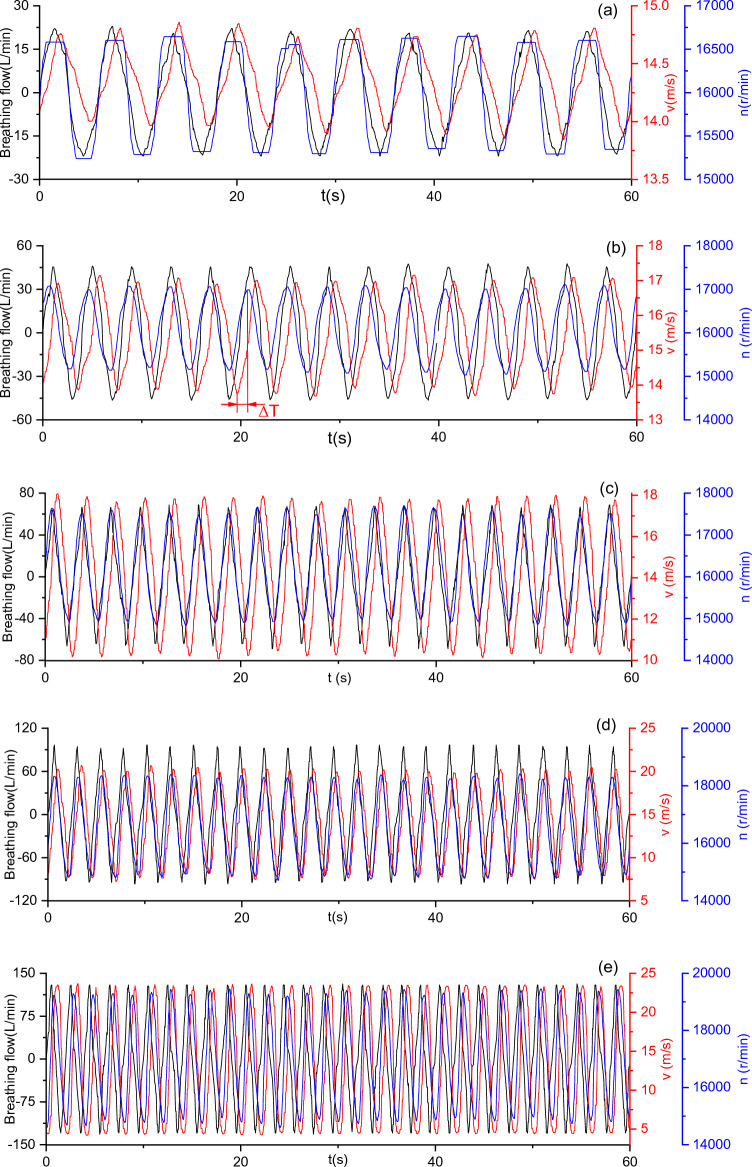
Variations of outlet air velocity and fan speed of BF-PAPR with breathing flow when $v_{s}$ = 14.6 m/s, *λ* = 1, and *m* = 0.

In the breath-following air supply experiment, the outlet air velocity and the fan speed under five different MIFs were experimentally researched with the aid of the human breathing simulator. It is found that the waveforms of both outlet air velocity and fan speed show the same variation trend, both varying periodically with the breathing flow. The waveforms of outlet air velocity and fan speed under the $v_{s}$, *λ,* and *m* values of 14.6 m/s, 1, and 0 respectively are exhibited in Fig. 6. According to Fig. 6, the waveform of the outlet air velocity is nearly sinusoidal. Besides, the fan speed varies approximately in a trapezoidal wave when the value of MIF is 15 L/min, while it varies in a sinusoidal wave when the value of MIF is 30–85 L/min. This phenomenon can be explained as follows: when the value of MIF is 15 L/min, the outlet air velocity changes insignificantly; resultantly, the fan speed does not change much after reaching its maximum and minimum, thus forming a trapezoidal wave. In an ideal situation, when the outlet air velocity reaches its minimum, the fan speed should be raised to the maximum to increase the air supply flow. However, delay would occur because it takes time for the fan speed to be raised and for the air to be transferred from the fan to the half mask.

### Effect of breathing flow on fan speed

**Figure 7:**
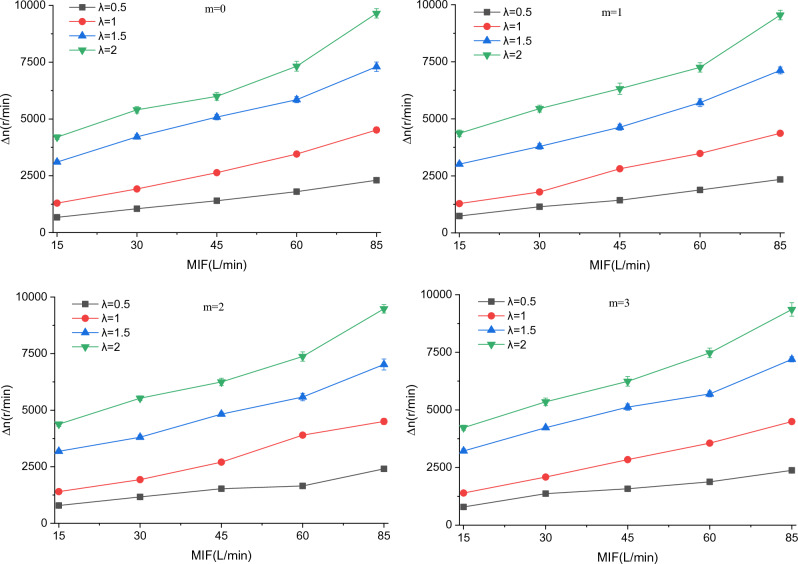
Variation of fan speed difference with MIF when $v_{s}$ = 14.6 m/s.

**Figure 8:**
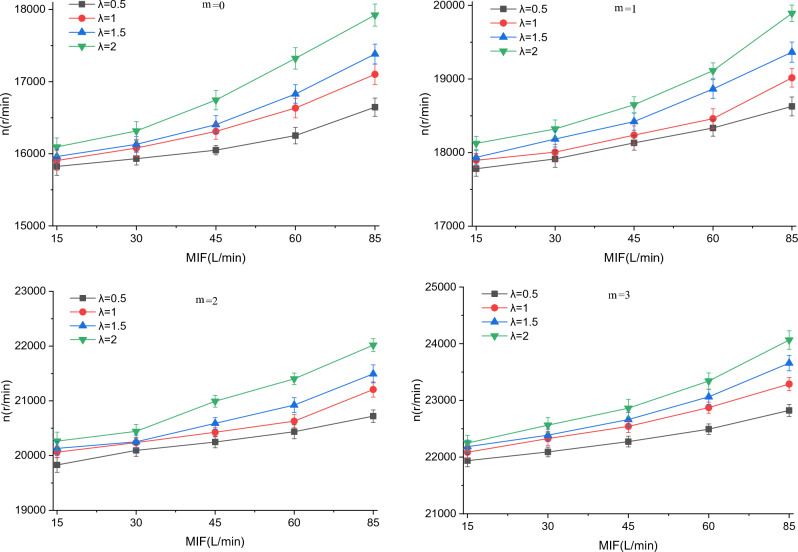
Variation of average fan speed with MIF when $v_{s}$ = 14.6 m/s.

Considering that the air supply of the BF-PAPR is related to the fan speed, the variations of fan speed difference (*∆n*) and average speed (*n*) with MIF were investigated. Since *∆n* and *n* vary in the same trend with MIF at different $v_{s}$ values (Supplementary Figs. S1 and S2), the variations of *∆n* and *n* with MIF at a $v_{s}$ value of 14.6 m/s are taken for analysis here (Figs. 7 and 8). It can be observed from Fig. 7 that *∆n* grows with the increases in MIF and *λ*, which is determined by the air supply flow adjustment model of the BF-PAPR. The increase in MIF will lead to a rise of the rate of change of $v_{hm}$, which will result in an increase in $\left| {v_{s} - v_{0} } \right|$. As can be known from Eq. (5), the variation of duty ratio (*∆D*) goes up as* λ* and $\left| {v_{s} - v_{0} } \right|$ increase, which leads to an increase in *∆n*. The increase in *∆n* conduces to the regulation of the air supply flow of the BF-PAPR, reducing the air supply flow during exhalation and raising the air supply flow during inhalation. It can also be seen from Supplementary Fig. S1 that *∆n* is essentially constant for different $v_{s}$ and *m*, i.e., $v_{s}$ and filtration resistance barely affect *∆n*.

For the same $v_{s}$ and *m*, the average fan speed (*n*) grows as MIF and *λ* increase (Fig. 8). The reason is that the increases in MIF and *λ* bring about a greater *∆n* which enables the fan to accelerate more quickly and decelerate more slowly. In addition, when *λ* is the same, *n* increases with the increases in $v_{s}$ and *m* (Supplementary Fig. S2), because the fan needs to speed up to provide more air supply flow and overcome the increased filtration resistance. The increase of* n* is inconducive to the reduction of the air supply flow of the BF-PAPR.

### Breath-following delay

**Figure 9:**
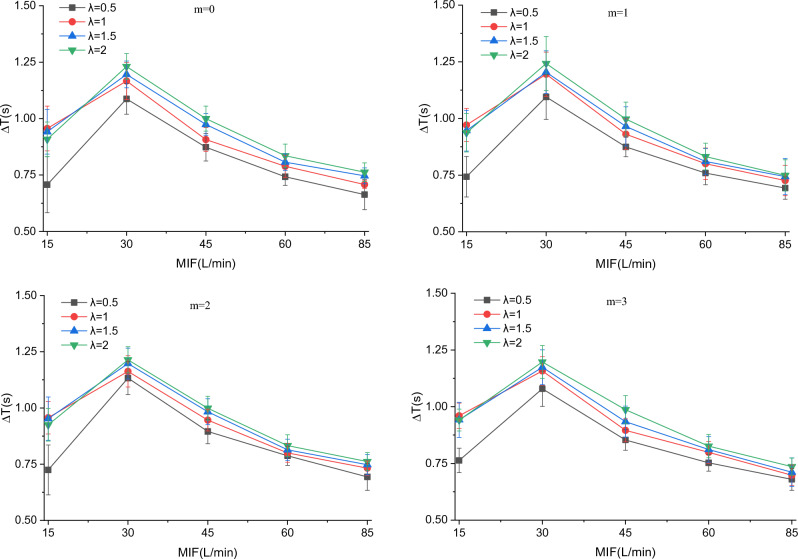
Variation of breath-following delay with MIF when $v_{s}$ = 14.6 m/s.

The breath-following delay $\Delta T$ was set as the time difference between the minimum outlet air velocity and the maximum fan speed. Since $\Delta T$ varies in the same trend with MIF at different $v_{s}$ values (Supplementary Fig. S3), the variation of $\Delta T$ with MIF at $v_{s}$ = 14.6 m/s is taken for analysis here (Fig. 9). As depicted in Fig. 9, $\Delta T$ falls with the increase in MIF in the MIF range of 30–85 L/min, which is caused by the shortening of the breathing cycle. It can also be seen that $\Delta T$ rises with *λ*, because at a larger *λ*, *∆n* will become greater and the fan needs a longer time to accelerate. At a MIF of 15 L/min, $\Delta T$ corresponds to different values, about 0.75 s when λ equals 0.5 and about 0.95 s when λ ranges from 1 to 2, as a result of the small variation of breathing flow at such a MIF. On the one hand, decreasing *λ* can reduce $\Delta T$, which facilitates the synchronization of air supply flow with breathing flow. On the other hand, it leads to a decrease in *∆n*, which hinders the adjustment of air supply flow with breathing flow.

### Effect of breathing flow on outlet air velocity

**Figure 10:**
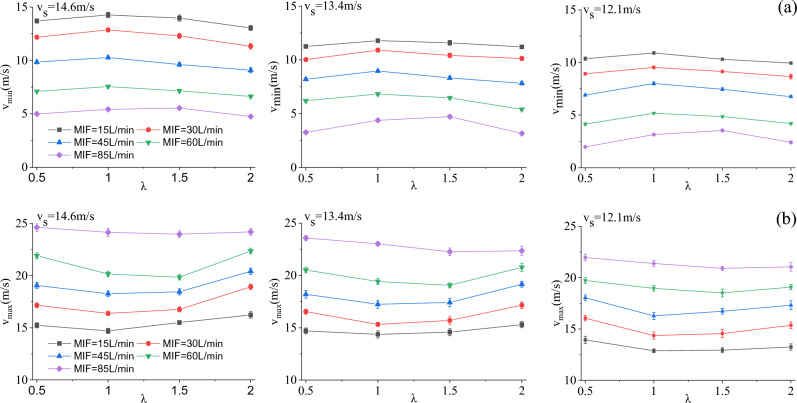
Variations of minimum and maximum outlet air velocities of the BF-PAPR with the duty cycle variation coefficient when *m* = 0.

In the breath-following air supply experiment, the minimum ($v_{\min }$) and maximum ($v_{\max }$) outlet air velocities under different experimental conditions were recorded. As disclosed by the experimental data, $v_{\min }$ and $v_{\max }$ remain essentially constant when different values are assigned to *m*. Hence, it can be concluded that *m* exerts no influence on the outlet air velocity, that is, the increase of filtration resistance barely affects the outlet air velocity. The variations of $v_{\min }$ and $v_{\max }$ of the BF-PAPR with λ are illustrated in Fig. 10. With the increase of *λ*, $v_{\min }$ increases first and then decreases, while $v_{\max }$ decreases first and then increases. In addition, when *λ* equals 1 or 1.5, $v_{\min }$ reaches its maximum and $v_{\max }$ reaches its minimum. This is attributed to the combined effect of fan speed difference and breath-following delay. To be specific, the fan speed difference becomes larger and the breath-following delay gets smaller at a *λ* value of 1 or 1.5. The increase of $v_{\min }$ responds to the increase of positive pressure inside the half mask, which can improve the respiratory protection effect of the BF-PAPR. Meanwhile, $v_{\min }$ and $v_{\max }$ decrease with the decrease in $v_{s}$ when MIF and *λ* are the same. The decrease in $v_{s}$ facilitates the reduction of the air supply flow of the BF-PAPR.

### Experimental research on constant air supply

**Figure 11:**
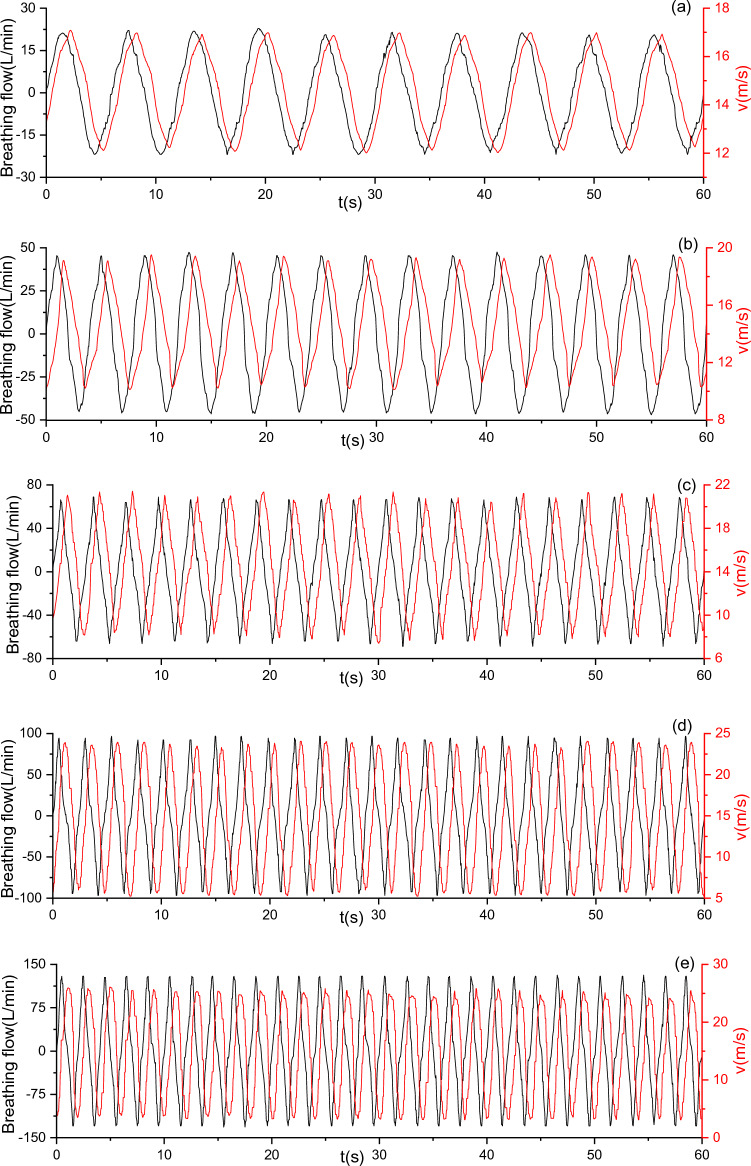
Variation of outlet air velocity with breathing under a constant air supply flow of 115 L/min (**a**) MIF = 15 L/min; (**b**) MIF = 30 L/min; (**c**) MIF = 45 L/min; (**d**) MIF = 60 L/min; (**e**) MIF = 85 L/min.

**Figure 12:**
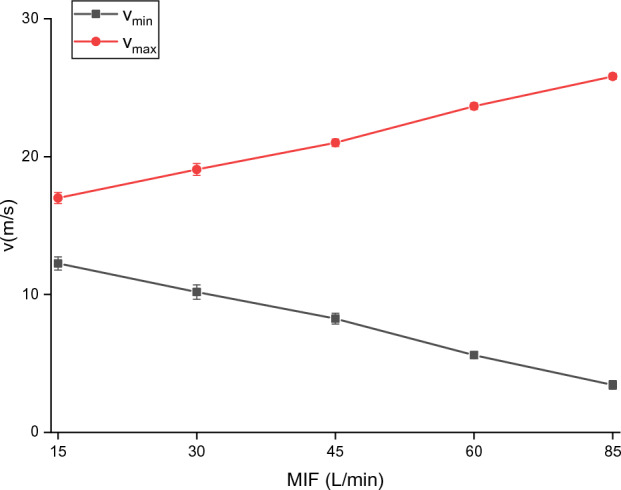
Variations of minimum and maximum outlet air velocities of the PAPR with MIF under a constant air supply flow of 115 L/min.

With a constant air supply flow of 115 L/min, the breathing flow and the outlet air velocity under five MIFs were measured (Fig. 11). As presented in Fig. 11, the waveform of outlet air velocity is approximately sinusoidal, and the larger the MIF, the wider the variation range of outlet air velocity. Furthermore, $v_{\max }$ and $v_{\min }$ within 1 min were recorded (Fig. 12). As shown in Fig. 12, $v_{\max }$ increases and $v_{\min }$ decreases with the increase in MIF, and the variation range of outlet air velocity keeps expanding. The PAPR can meet the needs of respiratory protection by providing sufficient air to maintain positive pressure in the half mask and keep a certain air velocity. Besides, it can be seen that $v_{\min }$ reaches its minimum, 3.41 m/s, when the MIF is 85 L/min.

### Minimum air supply flow of BF-PAPR

**Figure 13:**
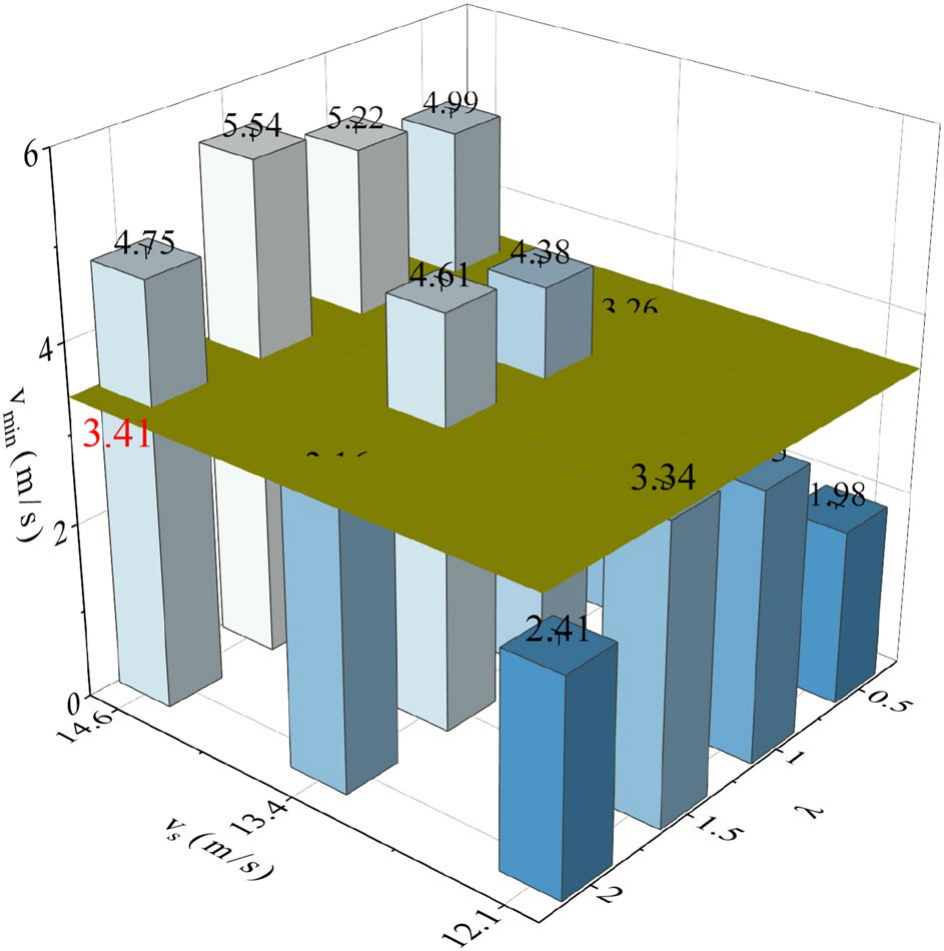
Minimum outlet air velocity of the BF-PAPR under the MIF of 85 L/min.

The PAPR meets the needs of respiratory protection by providing sufficient clean air, so the air supply flow under a varying MIF should be ensured. According to section "Experimental research on constant air supply", the minimum value of $v_{\min }$ is 3.41 m/s when the constant air supply flow is 115 L/min. That is to say, $v_{\min }$ should not be lower than 3.41 L /min for the purpose of guaranteeing the respiratory protection effect of the BF-PAPR. It is known that $v_{\min }$ reaches its minimum under the MIF of 85 L/min (Fig. 10), so the minimum outlet air velocity of the BF-PAPR under the MIF of 85 L/min is plotted in Fig. 13. As presented in Fig. 13, the $v_{\min }$ data are all lower than 3.41 m/s at a $v_{s}$ value of 12.1 m/s, which fail to meet the requirements of respiratory protection. From section "Effect of breathing flow on outlet air velocity", the decrease in $v_{s}$ facilitates the reduction of the air supply flow of the BF-PAPR. Thus, it can be concluded that the BF-PAPR can supply the least air flow while meeting the demand of respiratory protection when the value of $v_{s}$ is 13.4 m/s and the value of *λ* is 1 or 1.5.

**Figure 14:**
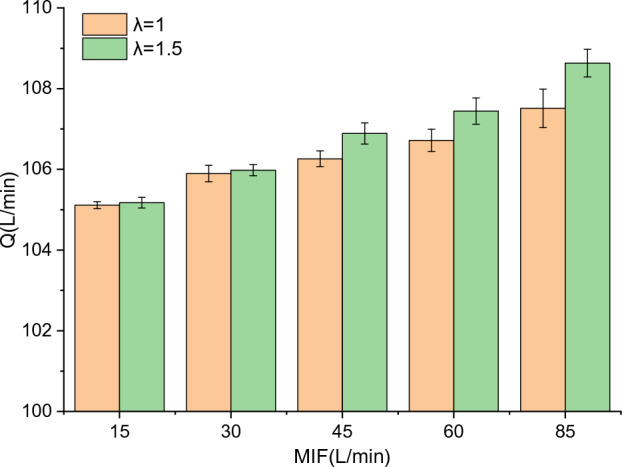
Variation of air supply flow with MIF when $v_{s}$ = 13.4 m/s.

In order to study the variation of the air supply flow of the BF-PAPR with the MIF, an electronic flowmeter was connected to the fan inlet (Fig. 5) for counting the air supply flow. The electronic flowmeter (Yamatake Azbil-MCFO150ARND) can monitor the air supply flow and count the total air supply volume. The air supply flow of the BF-PAPR at different MIFs is given in Fig. 14. It can be seen that the air supply flow is smaller when the value of *λ* is 1, due to the smaller average fan speed. Moreover, the air supply flow increases with the rise of MIF, because the average fan speed grows as the MIF rises. When the value of *λ* is 1, the air supply flows of the BF-PAPR at the MIFs of 15, 30, 45, 60, and 85 L/min are 105.1, 105.9, 106.3, 106.7, and 107.5 L/min, and the relative reductions are 8.6%, 7.9%, 7.6%, 7.2%, and 6.5%, respectively.

At the same working resistance, the relationship between the power of the fan and the air flow can be expressed as^31^:

7 $$\frac{{N_{1} }}{{N_{2} }}{ = }\left( {\frac{{Q_{1} }}{{Q_{2} }}} \right)^{3}$$

where $N_{1}$ and $N_{2}$ are the powers at the air flows of $Q_{1}$ and $Q_{2}$, respectively, W. The relative reduction in the power of the fan compared to a constant air supply flow of 115 L/min can be expressed as:

8 $$\gamma { = 1 - }\left( {\frac{{Q{\prime} }}{115}} \right)^{3}$$

**Figure 15:**
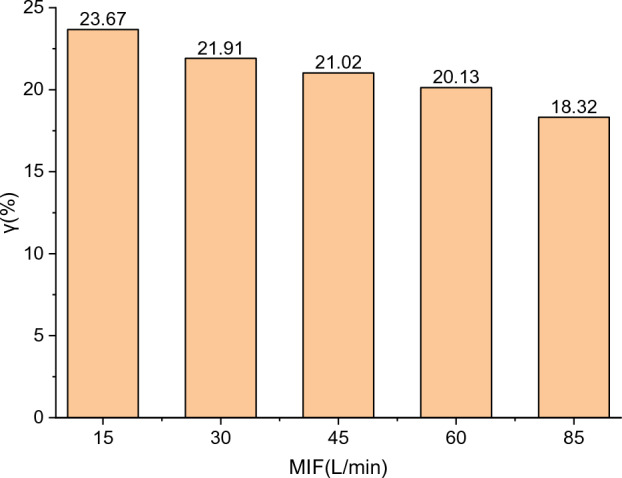
Power reduction of the BF-PAPR relative to the PAPR.

The relative reduction in the power of the BF-PAPR obtained from Eq. (8) is shown in Fig. 15. It can be seen that the power of the BF-PAPR at the MIFs of 15, 30, 45, 60, and 85 L/min is reduced by 23.67%, 21.91%, 21.02%, 20.13%, and 18.32% relative to that of the PAPR with a constant air supply flow of 115 L/min, respectively.

The BF-PAPR can reduce the air flow by 6.5%-8.6% compared to the PAPR while maintaining respiratory protection performance, which is beneficial for extending the service life of the filter and reducing the usage cost. At the same time, the BF-PAPR can reduce energy consumption by approximately 20%, which will help to extend its continuous working time. However, a BF-PAPR is more expensive than a traditional PAPR with a constant air supply flow. The breath-following adjustment system, consisting mainly of a single-chip microcontroller, a variable-frequency centrifugal fan, and a modular wind speed transmitter, costs about $200. The high price of the BF-PAPR may hinder its widespread use.

## Conclusions

The respirator that can adjust the air supply flow according to the breathing flow was studied, and the variations of fan speed, outlet air velocity and breath-following delay with MIF were explored. Specific conclusions are as follows:

1. According to the relationships between duty cycle and fan speed and between fan speed and air flow, the air supply flow adjustment model of the BF-PAPR is established. The model can realize the dynamic adjustment of the air supply flow of the BF-PAPR based on the breathing flow.
2. The fan speed difference increases with the duty cycle variation coefficient and the MIF, without being affected by the standard value of the air velocity and the filtration resistance. The increase of fan speed difference facilitates the adjustment of the air supply flow of the BF-PAPR.
3. At a MIF of 15 L/min, the breath-following delay changes insignificantly; at a MIF of 30–85 L/min, the breath-following delay increases with the coefficient of variation. The increase in breath-following delay is inconducive to the synchronization of air supply flow with breathing flow.
4. When the standard air velocity is the same, the minimum outlet air velocity increases first and then decreases with the increase of duty cycle variation coefficient, while the maximum outlet air velocity decreases and then increases.
5. When the standard value of the air velocity is 13.4 m/s and the duty cycle variation coefficient is 1, the air supply flow of the BF-PAPR reaches the minimum. The BF-PAPR can reduce the air supply flow by 6.5%-8.6% and the energy consumption by approximately 20% compared with the PAPR, which is beneficial for reducing the usage cost and extending the continuous working time.

## Supplementary Information

Supplementary Figures.

## Data Availability

The datasets used and/or analyzed during the current study are available upon reasonable request from the corresponding author.
