# Supplementary material for: Research on the air supply adjustment technology of breath-following powered air-purifying respirators
Source: Sci Rep. 2023 Jul 27;13:12219. doi: 10.1038/s41598-023-39411-z (PMC10374598; doi:10.1038/s41598-023-39411-z)
Supplement: Supplementary file 1 — Supplementary Figures. [file 41598_2023_39411_MOESM1_ESM.docx]

**Supplementary Figure S1**

Fig. S1 Variation of the speed difference of the fan with the MIF (a) m=0; (b) m=1; (c) m=2; (d) m=3

**Supplementary Figure S2**

Fig. S2 Variation of the average speed of the fan with the MIF (a) m=0; (b) m=1; (c) m=2; (d) m=3

**Supplementary Figure S3**

Fig. S3 Variation of breath-following delay with the MIF (a) m=0; (b) m=1; (c) m=2; (d) m=3
